# CONSORT in China: past development and future direction

**DOI:** 10.1186/s13063-015-0769-z

**Published:** 2015-06-01

**Authors:** Tian-Jiao Song, Hou-Fu Leng, Linda LD Zhong, Tai-Xiang Wu, Zhao-Xiang Bian

**Affiliations:** School of Chinese Medicine, Hong Kong Baptist University, Hong Kong, China; Chinese Cochrane Centre, Si Chuan University, Si Chuan, China

**Keywords:** Randomized controlled trials, Quality assessment, CONSORT Statement, Reporting guideline

## Abstract

The Consolidated Standards of Reporting Trials (CONSORT) Statement was published in 1996, and first introduced to China in 2001. Although CONSORT has been widely accepted in high-quality international journals, we still need to have more investigation on how many Chinese journals have adopted the CONSORT Statement, and whether the quality of reporting has improved. A systematic search of the “Instructions to authors” in all Chinese medical journals in China Academic Journals (CAJ) Full-text Database was conducted up to February 2012 and only 7 journals officially listed the requirements of the CONSORT Statement. The research articles about randomized controlled trials (RCTs) published in 2002, 2004, 2006, 2008, and 2010 from journals which had specifically adopted the CONSORT Statement, and from 30 top journals based on the Chinese Science Citation Index (CSCI) 2011 as the control group, were identified. The quality of both cohorts of articles was assessed using the revised CONSORT Checklist and Jadad scale. A total of 1221 Chinese medical journals was identified. Only seven journals stated clearly in the “Instructions to authors” that authors should adopt the CONSORT requirement in the clinical trial paper. None of these journals is among the control group in the CSCI 2011. In the selected years, a total of 171 articles from 7 journals which had adopted CONSORT and 232 articles in the control were identified as including RCT trials. The average scores according to the revised CONSORT Checklist were 29.47 for the CONSORT-adopting journals and 25.57 for the control group; while the average scores based on the Jadad scale were 2.53 for CONSORT-adopting journals and 1.97 for the control group. Few journals among Chinese medical journals have adopted the CONSORT Statement. The overall quality of RCT reports in the 7 journals which have adopted CONSORT was better than those in the top 30 journals which have not adopteded CONSORT. The quality of RCT reports in Chinese journals needs further improvement, and the CONSORT Statement could be a very helpful guideline.

## Background

Due to the unsatisfactory situation of randomized controlled trial (RCT) reporting, in 1993, 30 experts met in Ottawa, Canada with the aim of developing a new scale to assess the quality of RCT reports [1]. After merging with another group of experts from Chicago (USA), the Consolidated Standards of Reporting Trials (CONSORT) Statement was first published in 1996 [2]. Previous studies have shown that CONSORT did improve the quality of reports of RCTs [3, 4]. A revised CONSORT Statement was published in 2001 to refine this standard [5], and a further revision (CONSORT 2010 Statement) was published in 8 journals simultaneously in 2010 [6]. By 4 August 2011, 458 core international peer-reviewed medical journals on PubMed had endorsed the CONSORT Statement [6]. In China, a previous survey was conducted to assess the endorsement of the CONSORT Statement by high-impact medical journals in 2012 and found that only 6 journals mentioned “CONSORT” in their “Instructions to authors” [7].

Before 2007, the quality of RCT reports in China was not good. In an RCT quality evaluation based on the CONSORT 2001 Statement [8], 142 published RCTs from 2004 to 2007 in 5 leading Chinese medical journals were assessed and it was found that only a very small number of CONSORT items was reported clearly in all included trials. Another report, which included 307 RCTs conducted in China and published in 2004, came to a similar conclusion [9]. Many systematic review authors have pointed out that improving trial reporting quality was necessary if not also urgent [8, 9]. For the improvement of reporting quality, different parties, such as researchers, clinicians, journalists, funding agency, policy makers and drug companies, should work together to make sure that each paper meets certain minimum standards, thus ensuring that the public can access to pertinent trial information. In this circumstance, journalists, as the “door keepers” of publication, play a crucial role. From this point of view, the CONSORT Statement, providing specific and comprehensive guidelines for trial reporting, could be used by different parties as rules for good publication. Therefore, adoption of this standard becomes a very crucial step in informing the authors how to design, carry out and report trials of the best quality.

By 25 February 2012, 1221 medical journals were found in the database, and this number is expected to increase in the future. Among these journals, there are a few international peer-reviewed journals; for these journals, the low quality of the papers is always an issue for discussion [8, 9]. In this study, we firstly conducted a systematic search of the “Instructions to authors” in all Chinese medical journals to identify the journals adopting the CONSORT Statement. Then we compared these journals’ RCT report quality with a control group (top thirty highest-impact journals in China). Through these process we examined all the medical journals in China to explore how many of them had adopted the CONSORT Statement, how they used it, and the influence of the CONSORT Statement on the quality of papers published in these journals, to analyze the current application status of the CONSORT Statement in China and how to improve the statement’s acceptance in Chinese medical journals.

## Review

### Methods

#### Selection of Chinese medical journals

China Academic Journals (CAJ) Full-text Database is the most comprehensive, full-text database of Chinese journals in the world [10] (accessed from http://www.cnki.net). This database contains all the medical journals in China, both in English and in Chinese. We examined all the medical journals listed under the subtitle of “Journal Navigation” in the database [11].

#### Collecting “Instructions to authors”

“Instructions to authors” statements of each journal comprise the basic requirements for articles to be considered and/or accepted for publication in the journal. Therefore, “Instructions to authors” statements of each journal were collected and by checking these statements, it could be determined whether or not the journal endorsed the CONSORT Statement. The searching processes were as follows: firstly, the homepage of each journal was searched under the “Journal Navigation” in the CAJ database, then “Instructions to authors” was checked through the journal’s online website. Secondly, we searched the key words “author,” “information” and/or “instruction” and these were further investigated if no formal item of “Instructions to authors” could be found. Again, if no information for authors was found, the “Instructions to authors” statement for a journal was searched in the www.google.com, as a substitution.

#### Determining which journals adopted the CONSORT Statement

After downloading the “Instructions to authors,” eight students scrutinized the CONSORT Statement adoption in two individual groups. One group is for the journals which were identified as adopting the CONSORT Statement, and the other group is for the journals without any available “Instructions to authors.” TJ Song conducted a third check to confirm the results.

#### Journal selection for quality assessment of RCT

In order to assess the quality of reporting, trial articles were collected. The journals which had adopted the CONSORT Statement were selected as targets. The 30 top medical journals with the highest impact factor (IF) in the Chinese Science Citation Index (CSCI) 2011 were selected as the controls. There is no overlap between the top 30 journals in the CSCI system and the 7 journals adopting the CONSORT Statement; in other words, none of the top CSCI journals have adopted CONSORT.

#### Search strategy for RCT reports

##### Inclusion criteria

Based on the aim of this study, we would like to search all the RCT reports related to medication intervention. The following criteria for inclusion were set as follows. All trials had to meet all criteria:

i. Trials with interventions in human beings
ii. Trials with chemical agents, biological agents or Traditional Chinese Medicine (TCM) herbs or with acupuncture or electroacupuncture as interventions
iii. Trials with the aim of testing the therapeutic effects of interventions

The search strategy of RCTs is listed as below:

1. clinical observation.mp
2. clinical trial.mp
3. clinical study.mp
4. efficacy.mp
5. effectiveness.mp
6. 1 OR 2 OR 3 OR 4 OR 5 OR
7. random.mp
8. randomi*ed.mp
9. randomi*zation.mp
10. 7 OR 8 OR 9
11. 6 AND 11

##### Exclusion criteria

Articles reporting trials with nutritional therapy (oral feeding, iodine, and/or food) were excluded. Trials comparing the effects of surgery, psychological treatment, and/or physical therapies were also excluded.

##### Targeted RCT articles

**Fig. 1:**
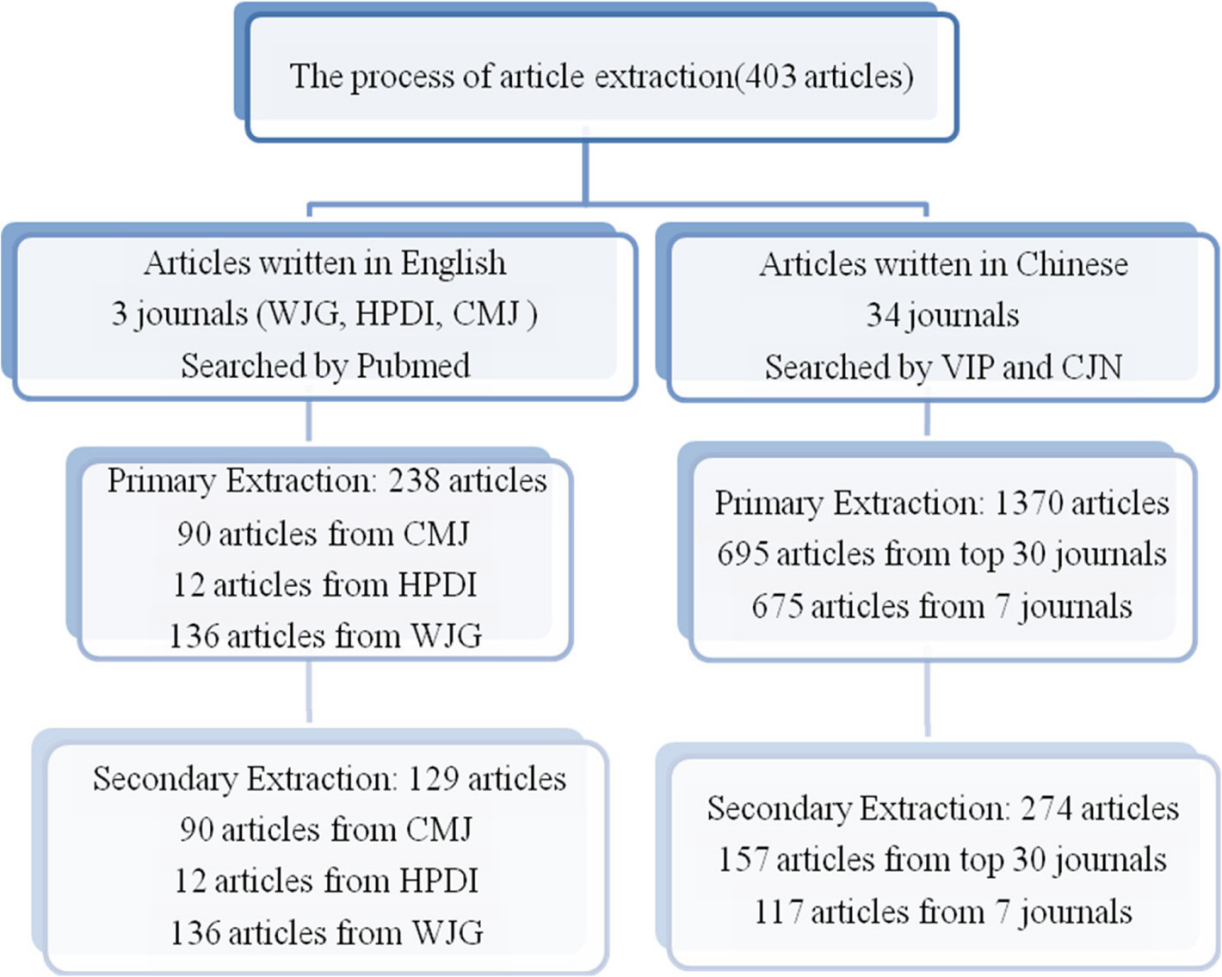
Flow chart of article search lists

Three databases were searched for articles meeting the inclusion criteria published in the selected journals. Articles written in Chinese were downloaded from the China Journal Net Database (http://www.cnki.net/) and Chinese Science and Technology Documents Database (VIP) (http://www.cqvip.com/). The search strategy was set. In order to limit the working load, it was decided, arbitrarily, to select only articles published in even numbered years; in other words, only articles published in 2002, 2004, 2006, 2008, and 2010 were counted. Duplicates from these two databases were removed. For articles written in English, PubMed (http://www.ncbi.nlm.nih.gov/pubmed/) was searched with the same limits regarding the publication year, i.e., 2002, 2004, 2006, 2008 and 2010. A flow chart of article search lists is presented in Fig. 1. Two reviewers (TJ Song and HF Leng) independently assessed the RCT articles.

#### Assessment of RCT report quality

##### CONSORT Checklist

The scoring scale was set based on the 2010 CONSORT Checklist in which there are 25 items [12]. In order to ensure that all items were included in the final trial report, each item with multiple contents was broken down such that each sub-item was counted separately, as listed in Table 1. Each extracted article was assessed using this scoring system. If the item was included in the RCT report, one point was awarded, and the total point score represented the quality of that clinical report. For quality assessment, firstly all reviewers underwent training in evaluating RCTs using the revised CONSORT Checklist, which including the statement and Explanation and Elaboration (E&E). During the assessment, discrepancies were resolved by consensus, or decided by ZX Bian. The score for each item was either 0 or 1 depending on whether the authors had reported it. The maximum score was 84. The scores for the 84 items were added together and a subgroup sum was conducted under the categories of Title (1), Abstract (2–7), Introduction (8–10), Methods (11–47), Results (48–71), Discussion (72–79), and Other Information (80–84).

**Table 1:** Revised 2010 Consolidated Standards of Reporting Trials (CONSORT) Checklist

Section/Topic	Number	Description
Title and Abstract	1	Identification as a randomized trial in the title
	2	Has an abstract
	3	Has a structured summary
	4	Summary including trial design
	5	Summary including methods
	6	Summary including results
	7	Summary including conclusions
Introduction		
Background and objectives	8	Scientific background
	9	Explanation of rationale
	10	Specific objectives or hypotheses
Methods		
Trial design	11	Description of trial design (such as parallel, factorial)
	12	Description including allocation ratio
	13	Important changes to methods after trial commencement (such as eligibility criteria)
	14	Reasons for changes to methods after trial commencement
Participants	15	Eligibility criteria for participants
	16	Settings and locations where the data were collected
Intervention	17	States precise details of the interventions intended for each group about how to conduct the administration which could allow replication
	18	States precise details of the interventions intended for each group about when to conduct the administration which could allow replication
Outcomes	19	Defined what is the primary outcome measures
	20	Completely defined how the primary outcome measures were assessed
	21	Completely defined when the primary outcome measures were assessed
	22	Defined what is the secondary outcome measures
	23	Completely defined how the secondary outcome measures were assessed
	24	Completely defined when the secondary outcome measures were assessed
	25	Any changes to trial outcomes after the trial commenced
	26	Reasons of changes to trial outcomes after trial commenced
Sample size	27	How sample size was determined
	28	When applicable, any interim analyses
	29	Explanation of the interim analyses
	30	When applicable, any interim stopping guidelines
	31	Explanation of stopping guidelines relative with interim analyses.
Randomization		
Sequence generation	32	Method to generate the random allocation sequence
	33	Types of randomization
	34	Details of any restriction for randomization (such as blocking and block size)
Allocation concealment mechanism	35	Mechanism used to implement the random allocation sequence (such as sequentially numbered containers)
	36	Describes any steps taken to concealed the sequence until interventions were assigned
Implementation	37	States who generated the allocation sequence
	38	States who enrolled participants?
	39	States who assigned participants to interventions (their trail groups)
Blinding	40	States that the trial is blinded or open.
	41	States how the trial is blinded
	42	States who was blinded after assignment to interventions (for example, participants, care providers, those assessing outcomes)
	43	If relevant, description of the similarity of interventions
Statistical methods	44	Defines the statistical methods used in the trail
	45	Defines statistical methods used to compare groups for primary outcomes
	46	Defines statistical methods used to compare groups for secondary outcomes
	47	Methods for additional analyses, such as subgroup analyses and adjusted analyses
Results		
Participant flow (a diagram is strongly recommended)	48	For each group, the numbers of participants who were randomly assigned
	49	For each group, the numbers of participants who received intended treatment
	50	For each group, the numbers of participants who were analyzed for the primary outcome
	51	For each group, losses and exclusions after randomization
	52	Reasons for losses and exclusions after randomization
Recruitment	53	Define the periods of recruitment
	54	Define the specific dates of recruitment
	55	Define the periods of follow-up
	56	Define the specific dates of follow-up
	57	Why the trial ended or was stopped
Baseline data	58	A table showing the baseline demographic and clinical characteristics for each group
Numbers analyzed	59	Actual number of participants in each group
	60	States whether the analysis was by original assigned groups
Outcomes and estimation	61	Summary of results for each group with primary outcomes
	62	Estimates effect size for primary outcomes
	63	Estimates precision of effect size (95 % confidence interval) for primary outcomes
	64	Summary of results for each group with secondary outcomes
	65	Estimates effect size for secondary outcomes
	66	Estimates precision of effect size (95 % confidence interval) for secondary outcomes
	67	For binary outcomes, presentation of both absolute and relative effect sizes is recommended
Ancillary analyses	68	Results of any other analyses performed, distinguishing prespecified from exploratory
	69	Results of subgroup analyses performed, distinguishing prespecified from exploratory
	70	Results of adjusted analyses performed, distinguishing prespecified from exploratory
Harms	71	All important harms or unintended effects in each group
Discussion		
Limitations	72	Trial limitations
	73	Addressing sources of potential bias
	74	Addressing sources of imprecision
	75	If relevant, addressing source of multiplicity of analyses
Generalizability	76	Generalizability (external validity or applicability) of the trial findings
Interpretation	77	Interpretation of results
	78	Balancing benefits and harms
	79	Considering other relevant evidence relating with the results
Other Information		
Registration	80	Registration number
	81	Name of trial registry
Protocol	82	Where the full trial protocol can be accessed, if available
Funding	83	Sources of funding and other support (such as supply of drugs)
	84	Role of funders

##### Jadad scale

The Jadad scale is a 5-point scale for evaluating the quality of randomized trials in which 3 points or more indicates superior quality [13, 14]. It is commonly used to evaluate RCT quality [15]. The Jadad scale includes 5 items: (1) description of randomization; (2) adequacy and appropriateness of randomization method; (3) description of single-blindness or double-blindness; (4) whether assessors are blinded to treatment conditions; and (5) description of withdrawals and drop outs. The score for each of the first 3 items was either 1 or 0 depending on whether the authors had reported it or not, respectively. The score for each of the last 2 items ranged from −1 to 1 (according to the explanation of the Jadad scale, −1 meant an inappropriate method description [16]). The maximum Jadad score was five. The scores for the five items were assessed by two independent persons, and the scores were added together. An average score was calculated.

Initially, they received the “*Revised CONSORT Statement for Reporting Randomized Trials: Explanation and Elaboration*” document, which provides the meaning and rationale for each checklist item and examples of good reporting practice. All reviewers received training in research methodology for basic clinical research methodology and evaluation of quality of RCT reports using Jadad scale provided by our taught course “Epidemiology and Critical Appraisal”, including the studied CONSORT Statement 2010. Besides, the assessment was blinded.

#### Data analysis

Data for descriptive statistics were analyzed using Microsoft Excel 2007 (Microsoft Inc., Redmond, WA, USA). For comparisons of reporting quality between the target group (seven journals) and control group (top thirty journals), an average score was calculated for each publication year studied.

### Results

#### Journals endorsing the CONSORT Statement until 2011

After searching for “Instructions to authors” on the CJN website and in its printed publication, 1140 official “Instructions to authors” were found in total. Further, 57 unofficial materials from websites other than the homepages of journals were collected. Also, 24 journals’ “Instructions to authors” could not be found.

After checking the content of “Instructions to authors”, only seven journals officially listed the requirements of the CONSORT Statement. These journals were as follows: *Chinese Journal of Cancer Biotherapy*, *Chinese Medical Journal* (*CMJ*, *published in English*), *Hepatobiliary & Pancreatic Diseases International* (*HPDI*, *published in English*), *Chinese Journal of Evidence-Based Medicine*, *Journal of Chinese Integrative Medicine*, *Shanghai Archives of Psychiatry*, and *Chinese Journal of Evidence-Based Pediatrics.*

#### Article extraction

Based on CSCI 2011, 30 top journals ranked by IF were chosen: these are listed in Table 2. After searching PubMed, VIP and CMJ (up to 19 January 2012), 1680 articles were extracted. Based on the exclusion criteria of this study, 403 articles were finally selected for further analysis, with 232 articles from the control group, and 171 articles from journals adopting the CONSORT Statement.

**Table 2:** Top 30 medical journals in the Chinese Science Citation Index 2011

Ranking	Name	Impact factor
1	*Chinese Journal of Interventional Imaging and Therapy*	1.4875
2	*Chinese Pharmacological Bulletin*	0.8357
3	*Chinese Herbal Medicine*	0.6875
4	*Journal of Pharmaceutical Sciences*	0.6674
5	*World Journal of Gastroenterology* (*WJG*)	0.6479
6	*The Journal of Immunology*	0.5564
7	*Chinese Journal of Endocrinology and metabolism*	0.5356
8	*Chinese Journal of Endemiology*	0.5337
9	*Chinese Journal of Pathophysiology*	0.5159
10	*Chinese Journal of Otology*	0.5116
11	*Chinese Journal of Virology*	0.5058
12	*Chinese Journal of Medical Imaging Technology*	0.5041
13	*Chinese Journal of Microsurgery*	0.4856
14	*Microbiology*	0.4444
15	*Acupuncture Research*	0.3864
16	*Chinese Critical Care Medicine*	0.3758
17	*China Journal of Chinese Materia Medica*	0.3655
18	*Acta Physiologica Sinica*	0.3598
19	*Chinese Journal of Pharmaceutical Analysis*	0.3458
20	*Chinese Journal of Neurology*	0.3380
21	*Chinese Journal of Preventive Medicine*	0.3290
22	*Chinese Journal of Radiation Oncology*	0.3265
23	*Acta Pharmacologica Sinica*	0.3204
24	*Chinese Traditional Patent Medicine*	0.3193
25	*Journal of Third Military Medical University*	0.3100
26	*Chinese Journal of Ultrasonography*	0.3052
27	*Chinese Journal of Nervous and Mental Diseases*	0.3043
28	*Chinese Journal of Reparative and Reconstructive Surgery*	0.2962
29	*Chinese Journal of Burns*	0.2953
30	*Chinese Journal of Antibiotics*	0.2953

#### Quality of articles

##### RCT quality assessed with the revised CONSORT 2010 Checklist

**Fig. 2:**
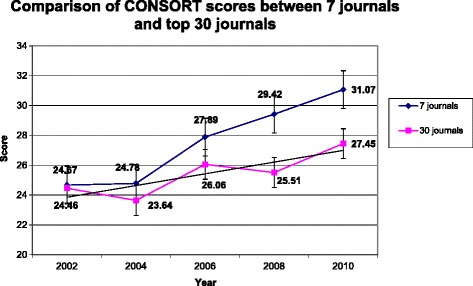
Randomized controlled trial (RCT) quality assessed with the revised Consolidated Standards of Reporting Trials (CONSORT) 2010 Checklist. For the top 30 journals, the scores for the 232 articles have slightly increased, and for the 7 journals which had endorsed the CONSORT Statement, the average score of article quality is slightly higher than that of articles from the top 30 journals

As shown in Fig. 2, the quality of RCT articles has increased from 2002–2010. For the top 30 journals, the scores for the 232 articles has slightly increased from 24.93 ± 3.51 in 2002, 23.78 ± 5.69 in 2004, 26.02 ± 5.93 in 2006 and 25.43 ± 5.50 in 2008 to 26.35 ± 5.94 in 2010. For the 7 journals which had endorsed the CONSORT Statement, the average score of article quality in 2002 was 25.46 ± 4.01, which is slightly higher than that of 24.93 ± 3.51 for articles from the top 30 journals, and the scores in the following years are also higher than that of the top 30 journals, with 27.97 ± 3.56 in 2004, 30.40 ± 5.16 in 2006, 29.22 ± 5.28 in 2008 and 31.29 ± 6.9 in 2010. The scores from the seven endorsing journals were better than those from the control group.

Furthermore, sub-item analysis showed that the reporting ratio for Abstracts and Introductions is high, with more than 80 % of CONSORT 2010’s suggested content being reported in both target and control groups of journals; however, the reporting rates of content in other sub-items were all below 50 %. The details were elaborated as follows:

##### Title and abstract

Among 171 articles from the target group of journals, 68 (40 %) identified their studies as RCTs in the title. Most articles provided abstracts; 5 did not (3 in 2004, 1 in 2006, and 1 in 2010). In contrast, only 8 % (19 out of 232) of the control group journals’ articles identified their studies as RCTs in the title, and 13 articles (1 in 2004, 6 in 2006, 4 in 2008, and 2 in 2010) did not have an abstract.

##### Introduction

The average scores of “Introduction” for 7 journals and the control group were 2.76 and 2.69, respectively. Every article from the 7 endorsing journals included an introduction, while 6 articles (2 from 2006, 3 from 2008, 1 from 2010) from the control group did not contain one.

##### Methods

**N**o article in any cohort showed a comprehensive method flow. Only 16.96 % (29 out of 171) of articles from the 7 endorsing journals and 7.33 % (17 out of 232) of articles from the control group clearly identified the type of randomization. Only 16.37 % (28 out of 171) of articles from the 7 journals and 6.47 % (15 out of 232) of articles from the control group gave a description of the allocation ratio. No article in either cohort of journals reported any important changes to methods after trial commencement. Nearly all the articles provided precise information about the eligibility criteria for participants; however, only 80.12 % (137 out of 171) of articles from the 7 endorsing journals and 50 % (116 out of 232) of articles from the control group provided precise details about the settings and locations where the data were collected.

For “Intervention,” the intervention delivery method was clearly presented, and 84.22 % (144 out of 171) of articles from the 7 endorsing journals and 95.26 % (221 out of 232) from the control group reported this information clearly. But, only 35.09 % (60 out of 171) of articles from the 7 endorsing journals and 34.91 % (81 out of 232) of articles form the control group provided precise time information.

For “Outcomes,” very few articles definitely stated the primary and secondary outcomes in their trial. Only 19.88 % (34 out of 171) of articles from the 7 endorsing journals and 9.05 % (21 out of 232) of articles from the control group mentioned it. Further, some articles neglected to report the assessment time and measurement for outcomes. No article reported any changes of trial outcome index.

For “Sample Size,” only 20 (15 from the 7 endorsing journals and 5 from the control group) articles reported how this was determined. No article gave any information about the interim analyses. Only 2 articles, from the *Chinese Journal of Evidence-Based Medicine* [17, 18], gave the interim stopping guideline of the whole trial.

For “Randomization,” “Allocation Concealment,” “Sequence Generation” and “Implementation,” no article could fulfill all requirements listed in the checklist. Moreover, in 33.92 % (58 out of 171) of articles from the 7 endorsing journals and 48.28 % (112 out of 232) of articles from the control group, they only mentioned “randomly” to represent all these items.

For “Blinding,” only 32.75 % (56 out of 17z1) of articles from the 7 endorsing journals and 21.12 % (49 out of 232) of articles from the control group stated whether the trial was blinded or open. No article got full marks in this area. In all 403 articles, only 46 described how to conduct blinding.

For “Statistical Methods,” 96.53 % (389 out of total 403) of articles defined the statistical methods used in the trial. Nevertheless, only 8 articles (5 from the 7 endorsing journals and 3 from the control group) pointed out the statistical methods used in comparing primary or secondary outcomes. Four articles (two from the seven endorsing journals and two from the control group) pointed out the statistical methods used in additional analysis.

##### Results

No significant superiority was found for articles from the 7 endorsing journals for “Results,” with the average score 8.36, comparing with 7.03 for articles from the control group. For “Participant flow,” almost all the articles (98 % from the 7 endorsing journals and 97.84 % from the control group) reported the numbers of participants who were randomly assigned. However, the percentage of articles that reported the numbers of participants who had received intended treatment was less: 22 % (37 out of 171) for the 7 endorsing journals and 9 % (20 out of 232) for the control group. Fifty percent (86 out of 171) of articles from the 7 endorsing journals and 31.03 % (72 out of 232) of the articles from the control group clearly reported the number of losses and exclusions after randomization. However, only 42 % (44 out of 171) of articles from the 7 endorsing journals and 21.98 % (51 out of 232) of articles from the control group gave the reasons. For “Recruitment,” as high as 77.78 % (133 out of 171) of articles from the 7 endorsing journals and 68.97 % (160 out of 232) of articles from the control group defined the periods of recruitment. Among them, 9 out of 133 articles and 17 out of 160 articles neglected the specific month. Around half the articles (51.46 % from the 7 endorsing journals and 48.28 % from the control group) reported the follow-up periods. Only ten articles (two from the seven endorsing journals and eight from the control group) stated the specific dates of follow-up. Seven (five from the seven endorsing journals and two from the control group) articles in total reported the reason why the trial ended or was stopped. For “Baseline Data,” 38.01 % (65 out of 171) of articles from the 7 endorsing journals and 34.48 % (80 out of 232) of articles from the control group showed the baseline demographic and clinical characteristics for each group. For “Number Analysis,” 24.56 % (42 out of 171) of articles from the 7 endorsing journals and 10.34 % (24 out of 232) of articles from the control group explained how the analysis was performed, either “intention-to-treat” or “per protocol.”

For “Outcomes and Estimation items,” if the articles identified what the primary and secondary outcomes were, almost all of them would summarize the results separately. Very limited numbers of articles estimated the effect size between groups. No articles published in 2002 estimated effect size for both primary and secondary outcomes together with precision. Less than 5 % of articles for both the 7 endorsing journals and the control group reported all the 4 points related to effect size and precision of effect size (62, 63, 65, and 66). No binary outcome was presented of both absolute and relative effect size. For “Ancillary Analyses,” only 2 articles (1 from the 7 endorsing journal group and 1 from the control group) published in 2010 mentioned other analyses which were performed, distinguishing prespecified from exploratory. The article from the control group also mentioned the adjusted analyses which were performed, also distinguishing prespecified from exploratory. For “Harms,” 92.98 % (159 out of 171) articles of the 7 endorsing journals reported important harms or unintended effects in each group, whereas, only 37.5 % of articles from the control group reported it.

##### Discussion

For “Limitations,” no article clearly separated potential bias with imprecision, though some articles (32.75 % for the 7 endorsing journals and 13.36 % for the control group) mentioned that their trials had limitations. No relevant source of multiplicity analyses was addressed for all the articles. For “Generalizability,” only one fifth of articles (29.82 % for the 7 endorsing journals and 18.97 % for the control group) mentioned it. For “Interpretation,” almost all articles (400 out of 403) interpreted the results except 3 articles (1 from the 7 endorsing journal group and 2 from the control group). The majority of articles (71.35 % for the 7 endorsing journals and 64.22 % for the control group) considered other relevant evidence relating with the results.

##### Other information

Nearly half the articles (41.52 % for the 7 endorsing journals and 43.10 % for the control group) provided sources of funding and other support, but only 4 articles (3 from the 7 endorsing journals and 1 from the control group) identified the role of funders. Only seven articles (five from the seven endorsing journals and two from the control group) mentioned trial registration number, six of them gave the name of trial registry (four from the seven endorsing journals and two from the control group). No article mentioned where the full trial protocol could be accessed.

##### The score assessed by the Jadad scale

**Fig. 3:**
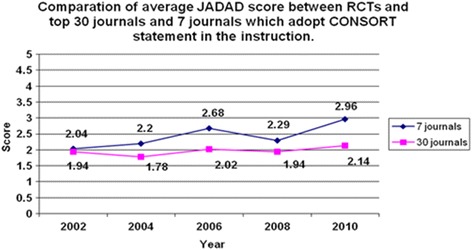
The score assessed by the Jadad scale. The tendency of the Jadad score was in accord with that of the Consolidated Standards of Reporting Trials (CONSORT) score

The range of the Jadad score for all the articles was between 0 and 5. As shown in Fig. 3, the tendency of the Jadad score was in accord with that of CONSORT score. For the 7 journals which had endorsed the CONSORT Statement, the average score for the 171 articles increased from 2.04 in 2002, 2.2 in 2004, 2.68 in 2006, and 2.29 in 2008 to 2.96 in 2010. For the control group, the average score for the 232 articles has been slightly increased from 1.94 in 2002, 1.78 in 2004, 2.02 in 2006 and 1.94 in 2008 to 2.14 in 2010.

For item 1 of Jadad scale, all the articles got a full mark. Although some articles did not identify the name of the trial design, the use of words such as “randomly,” “random,” and “randomization” could be found in all the articles. For item 2 of Jadad scale, only about one fifth of the RCTs (28.07 % from the 7 endorsing journal group versus 17.67 % from the control group) were designed as double-blinded trials. For item 3 of Jadad scale half the articles (53.80 %) from the 7 endorsing journals reported the drop-out situation, whereas, only 29.74 % of articles from the control group reported it. For item 4 of Jadad scale although articles reporting the method for randomization from both cohort journals could reach about 50 %, a total of 8 articles reported a wrong method according to the definition of inappropriate randomization method from the Jadad scale. For item 5 of Jadad scale, about 10 % of articles (15.20 % from the 7 endorsing journals and 7.76 % from the control group) reported the double-blind method. No apparently inappropriate double-blind method was found for this item.

### Discussion

The fact that China publishes 1221 medical journals in total indicates the large scale of its medical journal output. Clearly, some journals published “Instructions to authors” in the Announcement section and updated these frequently; but some journals had not updated their “Instructions to authors,” for more than 10 years. Further, the content of “Instructions to authors” varies considerably; the simplest one is one sentence only, which amounts to “key theme, clear statement, accurate evidence and standard writing.” It is evident that “Instructions to authors” constitutes a bridge between the authors and journalists, and the journal in question can tell the authors what kind of research articles they want and what kind of standard the article should meet. The current situation regarding “Instructions to authors” may reflect the observation that not all journals could be improved upon the quality of the articles published in their journals Besides, most of the journals only mentioned the CONSORT Statement and did not point out the version of CONSORT Statement. In these instances, perhaps the translation of the CONSORT Statement was conducted by the editors without any verification process. On the other hand, even the journals that had adopted CONSORT 2010 were those that routinely provide information to authors about good reporting so this was already happening before the translation of that statement.

CONSORT Statements were first published in 1996 but not formally introduced to China until they appeared in 2 articles published in October 2001 [17, 18]; formal full translation of the CONSORT Statement endorsement by the CONSORT Group was in October 2007 [6]. However, based on our search results, only 7 Chinese medical journals had introduced the CONSORT Statement in their “Instructions to authors” up to February 2012. These figures imply that CONSORT has been largely ignored in China since its publication.

Strong evidence, however, has shown that CONSORT has improved the quality of reporting [3, 4]. Results from evaluating papers using the CONSORT Checklist and the Jadad scale showed that the quality of RCT reports published in Chinese medical journals has generally improved in the past ten years; even so, the level of quality is worrisome. The average scores of the articles from two cohort journals were low. This result is consistent with that from other researchers [8, 19–21]. Encouragingly, the quality of articles published in the 7 journals which have adopted the CONSORT Statement is higher than that of the top 30 journals. These data suggest that CONSORT endorsement contributes to this slight difference and is also consistent with studies of journals published in other parts of the world. For example, a review in 2001 showed that the quality of RCTs from Western journals which adopted CONSORT (*BMJ, JAMA* and *The Lancet*) is higher than that from *The New England Journal of Medicine*, which did not adopt CONSORT at that time [3]. Another report published in 2006 also proved that CONSORT guideline could improve RCT quality [22] and Chinese researchers now acknowledge that adoption of CONSORT leads to an improvement in report quality [8, 19].

On the other hand, the quality of RCTs from the target cohort of journals is not as high as we expected. The real, or absolute, quality of them was unsatisfactory. The same conclusion has been drawn from other reports. A typical example is an article from the *Journal of Chinese Integrative Medicine* [23], which clearly stated that CONSORT requirements were followed, but its CONSORT Checklist score is only 38 (45.24 %). CONSORT endorsement is only one step toward improving journal article quality. Other factors, such as the training background of researchers, the original design of the trial, requirements of journal, etc., are highly related to the final quality of reporting. Improving the design, execution and reporting of RCTs is a systematic task with multiple aspects. Because the CONSORT Statement addresses all of these aspects, we recommend that it should be implemented in full as a reporting standard.

### Limitations

The systematic search strategy was set first to find articles meeting the inclusion criteria. The article search was limited to two databases (CJN and VIP). Although these two databases cover most medical journals in China, there is a possibility that we missed some data. The likelihood that the results of using CONSORT are associated with better quality of clinical trials’ outcomes is also discussed in order to spread and recommend this practice among Chinese medical journal editors.

## Conclusion

Relatively few medical journals in China have adopted the CONSORT Statement. After assessing RCTs using both the detailed CONSORT Checklist and the Jadad Checklist, results showed that the overall quality of reports of RCTs in the 7 journals which had adopted the CONSORT Statement was better than those in the top 30 journals. Nevertheless, the overall quality of RCT reports in journals of China, though increasing, was poor.

### Recommendations

The CONSORT Statement is a valuable effort by researchers to standardize the reporting of trials. Results prove that this statement really helps improve RCT trial design, conducting and reporting. For this reason, we highly recommended that: 1) all journals should present clear and concrete “Instructions to authors” in readily accessible ways; and 2) each medical journal in China should adopt the CONSORT Statement.

## Abbreviations

CONSORT: The Consolidated Standards of Reporting Trials
CAJ: China academic journals
RCTs: Randomized controlled trials
CSCI: The Chinese science citation index
IF: Impact factor
TCM: Traditional chinese medicine
E&E: Explanation and elaboration
CMJ: Chinese medical journal
HPDI: Hepatobiliary & pancreatic diseases international

## References

[1] Moher D. CONSORT: how CONSORT began. http://www.consort-statement.org/about-consort/history. Accessed on Jan. 12nd, 2015.

[2] Begg C, Cho M, Eastwood S (1996). Improving the quality of reporting of randomized controlled trials. The CONSORT statement. JAMA.

[3] Moher D, Jones A, Lepage L (2001). Use of the CONSORT statement and quality of reports of randomized trials: a comparative before and after evaluation?. JAMA.

[4] Egger M, Jüni P, Bartlett C (2001). The value of patient flow charts in reports of randomized controlled trials: bibliographic study. JAMA.

[5] Moher D, Schulz KF, Altman DG (2001). The CONSORT statement: revised recommendations for improving the quality of reports of parallel group randomized trials. BMC Med Res Methodol.

[6] Moher D. CONSORT: development of CONSORT 2010. http://www.consort-statement.org/about-consort/history. Accessed on Jan. 12nd, 2015.

[7] Li XQ, Tao KM, Zhou QH, Moher D, Chen HY, Wang FZ (2012). Endorsement of the CONSORT Statement by high-impact medical journals in China: a survey of instructions for authors and published papers. PLoS One.

[8] Xu L, Li J, Zhang M, Ai C, Wang L (2008). Chinese authors do need CONSORT: reporting quality assessment for five leading Chinese medical journals. Contemp Clin Trials.

[9] Zhang D, Yin P, Freemantle N, Jordan R, Zhong N, Cheng KK (2008). An assessment of the quality of randomised controlled trials conducted in China. Trials.

[10] China Academic Journal Electronic Publishing House. China Academic Journals Full-text Database Description. http://www.cnki.net (Accessed on 16 June 2012).

[11] Ren LJ (2005). Comparison of five China citation databases. Inform Sci.

[12] Kenneth FS, Douglas G, Moher D, for the CONSORT Group (2010). CONSORT 2010 Statement: updated guidelines for reporting parallel group randomized trials. Ann Intern Med.

[13] Jadad AR, Moore RA, Carroll D (1996). Assessing the quality of reports of randomized clinical trials: is blinding necessary?. Control Clin Trials.

[14] Balasubramanian SP, Wiener M, Alshameeri Z (2006). Standards of reporting of randomized controlled trials in general surgery: can we do better?. Ann Surg.

[15] Kiehna EN, Starke RM, Pouratian N (2010). Standards for reporting randomized controlled trials in neurosurgery. J Neurosurg.

[16] Xiao HT, Zhong L, Tsang SW, Lin ZS, Bian ZX (2015). Traditional Chinese medicine formulas for irritable bowel syndrome: from ancient wisdoms to scientific understandings. Am J Chin Med.

[17] Liu YX, Yao C, Chen F, Yang YC (2001). Introduction of CONSORT statement of reporting randomized controlled clinical trials. Natl J Androl.

[18] Editorial Office of Chinese J of EBM (2001). Revised CONSORT: a suggestion to improve the reporting quality of RCT. Lancet.

[19] Wang L, Li Y, Li J, Zhang M, Xu L, Yuan W (2010). Quality of reporting of trial abstracts needs to be improved: using the CONSORT for abstracts to assess the four leading Chinese medical journals of traditional Chinese medicine. Trials.

[20] Wang G, Mao B, Xiong ZY, Fan T, Chen XD, Wang L (2007). CONSORT Group for Traditional Chinese Medicine. The quality of reporting of randomized controlled trials of traditional Chinese medicine: a survey of 13 randomly selected journals from mainland China. Clin Ther.

[21] Jia CY, Wang YC, Bai F (2006). Analysis of the quality of papers dealing with clinical trials in “Chinese Journal of Burns” during 2000–2004 by the standard of evidence-based medicine. Zhonghua Shao Shang Za Zhi.

[22] Kane RL, Wang J, Garrard J (2007). Reporting in randomized clinical trials improved after adoption of the CONSORT statement. J Clin Epidemiol.

[23] Chang J, Zhang RM, Zhang Y, Chen ZB, Zhang ZM, Xu Q (2008). Andrographolide drop-pill in treatment of acute upper respiratory tract infection with external wind-heat syndrome: a multicenter and randomized controlled trial. Zhong Xi Yi Jie He Xue Bao.

